# In vitro comparison of viral replication and cytopathology induced by SARS-CoV-2 variants

**DOI:** 10.1099/acmi.0.000716.v3

**Published:** 2024-07-22

**Authors:** Kruttika S. Phadke, Nathaniel B. A. Higdon, Bryan H. Bellaire

**Affiliations:** 1 Veterinary Microbiology and Preventive Medicine, Iowa State University, Ames 50011, USA; 2 Interdepartmental Microbiology Graduate Program, Iowa State University, Ames 50011, USA

**Keywords:** A549, Calu3, cytopathic effect (CPE), SARS-CoV-2, vero E6, viral replication, BSL3

## Abstract

A myriad of coronaviruses cause diseases from a common cold to severe lung infections and pneumonia. SARS-CoV-2 was discovered to be the etiologic agent of the Coronavirus pandemic and many laboratory techniques were examined for virus culture and basic and applied research. Understanding the replication kinetics and characterizing the effect the virus has on different cell lines is crucial for developing *in vitro* studies. With the emergence of multiple variants of SARS-CoV-2, a comparison between their infectivity and replication in common cell lines will help give us a clear understanding of their characteristic differences in pathogenicity. In this study we compared the cytopathic effect and replication of Wild-Type (USA/WA1), Omicron (B.1.1.529), and Delta (B.1.617.2) variants on five different cell lines; VeroE6, VeroE6 cells expressing high endogenous ACE2, VeroE6 cells expressing human ACE2 and TMPRSS2, Calu3 cells highly expressing human ACE2 and A549 cells. This data will aid researchers with experimental planning and viral pathogenicity analysis and provide a baseline for testing any future variants.

## Data Summary

Supporting data for *in vitro* comparison of SARS-CoV-2 variants deposited at AccessMicrobiology.com/figshare (https://doi.org/10.6084/m9.figshare.24246442.v1). These are the data files for the percent cell viability figures in the paper [1].

## Introduction

Coronaviruses from the Coronaviridae family, are enveloped RNA viruses that can infect humans and animals [2]. The most recently discovered coronavirus, SARS-CoV-2, was first identified in Wuhan, China in late 2019 and was declared a pandemic in 2020 by the World Health Organization (WHO) [3,4]. This virus causes the disease COVID-19, which has spread all over the world and is estimated to be responsible for almost 700 million cases and 7 million deaths [5] It is primarily a respiratory virus that is transmitted mainly through aerosols causing symptoms like fever, cough, fatigue and pneumonia and respiratory distress in more severe cases [6,8].

Throughout the pandemic, many variants of SARS-CoV-2 have emerged from different regions of the world that have quickly become a cause of concern. Two of these variants are Delta (B.1.617.2) and Omicron (B.1.1.529). Delta variant was first identified in late 2020 and is on the Variants Being Monitored (VBM) list by the Centres for Disease Control and Prevention (CDC) [9]. It consists of almost 30 mutations that contribute towards its increased infectivity and transmissibility, making it more dangerous than the Wild-Type (WT) [10,11]. Omicron variant was first identified in November 2021 and is currently the variant of concern (VOC) by the CDC [9]. After sequencing, it was identified that this variant has almost 50 mutations, most of which are in the receptor binding domain of the spike protein. Due to its high transmissibility and ability to evade neutralizing antibodies, it rapidly made its way around the globe [12].

During investigations into the pathogenesis of coronavirus variants, it became increasingly difficult to compare results with SARS-CoV-2 variants given their specific dependence on host cell types for replication. A relative comparison of *in vitro* characteristics such as cellular infectivity and sensitivity to neutralizing antibodies would be beneficial to address these difficulties. The lower cellular infectivity of recent variants confounds such a comparison since they require stable cell lines overexpressing cell receptors for routine culture. As a result, using different cell lines complicates the analysis of cellular assays such as relative infectivity, virus replication rates, and antibody neutralization changes between WT and variant viruses.

We report our observations on the relative infectivity and replication of the WT, Omicron, and Delta variants in multiple cell lines to identify characteristics specific to each variant. For *in vitro* studies such as antiviral testing, live virus neutralization assays and cytopathic effect (CPE) based assays, understanding the infection kinetics of the variants in the VeroE6 cell line is essential. VeroE6 cells express a high amount of the SARS-CoV-2 receptor, ACE2, on its surface, which makes it the ideal cell model for *in vitro* analysis [13,14]. TMPRSS2 is the protease essential for viral Spike protein cleavage and viral entry [13,15]. Enhancing the expression of both ACE2 and TMPRSS2 can give us a better understanding of their importance in the viral infection cycle of the variants. Here we have compared all three variants in VeroE6 cells and VeroE6 cells that are overexpressing ACE2 and TMPRSS2. Lastly, the variants are also tested in the more physiologically relevant human lung cell lines, Calu3 and A549s.

## Methods

### Biohazard statement

All infection experiments involving SARS-CoV-2 were performed in BSL-3 laboratory facilities at Iowa State University (ISU) with Institution Biosafety Committee approved protocols.

### Virus strains, cell culture, and virus amplification

African Green Monkey tissue culture cell line, Vero E6 (ATCC CRL-1586) cells and human lung epithelial, A549 cells (ATCC CCL185) were acquired from ATCC (Manassas, VA). VeroE6 high expressing human (h)ACE2 (NR53726) (VeroE6/ACE2), VeroE6 expressing TMPRSS2 and hACE2 (NR54970) (VeroE6/hACE2/TMPRSS2) (JCRB Cat# JCRB1819, RRID:CVCL_YQ49) and Calu3 high expressing human ACE2 (NR55340) (Calu3/hACE2) were acquired from BEI (Manassas, VA). All cells were maintained in Dulbecco’s modified Eagle medium (DMEM) (Corning) containing 10 % fetal bovine serum (FBS) (Cytiva). Wild-Type (USA/WA1) SARS-CoV-2 (NR-52281) isolated from a COVID-19 patient in Washington, USA, Delta strain B.1.617.2 (NR-55671) and Omicron strain B.1.1.529 (NR-56481) were acquired from BEI. Viral infectious doses were prepared from supplied stocks by amplifying the virus through three passages at which time aliquots were prepared and frozen. TCID50 and PFU quantification of the stocks were conducted on VeroE6/hACE2/TMPRSS2 by Spearman and Karber algorithm [16]. All TCID50 were converted to PFU using the following formula: PFU ml^−1^=0.7 × TCID50 ml^−1^ (https://www.atcc.org/resources/culture-guides/virology-culture-guide). Wild-type virus was amplified with Vero E6 cells and Omicron and Delta variants were amplified with the hACE2/TMPRSS2 overexpressing VeroE6 cell line. Regarding the potential of polybasic furin mutations in WT virus passaged in VeroE6 cell lines, stocks from passage three were also used to infect Syrian hamsters where the characteristic weight loss was observed (data not shown).

### Experimental infections

Cell cultures were seeded at a density of 2×10^4^ cells per well in 96-well plates 18 h prior to infection. Viral stocks were thawed and diluted serially from 1000 PFU to 0.01 PFU in DMEM with 5 % FBS. Infection was initiated by replacing media on the cells with 100 µl DMEM with 5 % FBS per well and adding 100 µl of each dilution to the cells. Each infection dilution was performed in triplicate. Infected cells were incubated for 5 days at 37 °C under 5 % CO_2_ atmosphere and cells were observed under an inverted microscope every day. On day 5, the LDH levels were measured from 50 µl of each cell culture supernatant using the CyQuant LDH Cytotoxicity Assay (Invitrogen C20301) according to the manufacturers’ protocol. Cell viability was calculated using the lactate dehydrogenase enzyme (LDH) released into the supernatant from infected cells at 490 nm and 680 nm. Cell Viability Percentage = 1-[(A_490_-A_680_)-Average of A_Uninfected(490-680)_].

Negative percent viability values are plotted as zero for figures shown. Viral enumeration of 100 µl of the culture supernatants from each well was done by RT-qPCR.

### RNA isolation and RT-qPCR analysis

Viral genome equivalents were quantified from RNA harvested from cell culture supernatants. TRIzol reagent (Sigma) was used to extract RNA by mixing 400 µl of TRIzol reagent with 100 µl of cell culture supernatant. Nucleic acids were separated by the addition of 80 µl of chloroform followed by centrifugation at 12 000 ***g*** at 4 °C for 15 min. RNA was precipitated by adding 200 µl of isopropanol to the upper aqueous layer and centrifuging for 10 min at 12 000 ***g*** and 4 °C. RNA pellet was washed with ethanol and resuspended in 50 µl of nuclease-free water. RNA concentrations were normalized to 50 ng µl^−1^. RT-qPCR of the extracted RNA was carried out using Luna Universal Probe One-Step RT-qPCR Kit and IDT 2019-nCoV RUO Kit on the Bio-Rad CFX96 Real-Time system. Cycle quantification (Cq) values were used to make graphs.

### Statistical analysis

Data analysis including calculations of average of means, standard deviation, or standard error and two-way ANOVA with a Bonferroni’s comparison of means was calculated using GraphPad Prism (Version 9.4.1, RRID:SCR_002798) GraphPad Software, San Diego, CA, USA. Negative percent viability values are plotted as zero for the figures shown. Viral titre was calculated by RT-qPCR using cycle quantification (Cq) values that reflect the change in cycles needed to detect viral RNA for the SARS-CoV-2 N1 protein.

## Results

A benchmark permissive cell line for many viruses, including members of the coronaviradae, are the epithelial-like, African green monkey kidney cell line known as Vero E6, subcultured from original Vero cells in 1979. Vero E6 cells continue to be used for *in vitro* analysis of SARS-CoV-2 propagation, viral pathogenesis, pharmacology, diagnostics, and immunology research. The inability of Vero E6 to produce interferon following viral infection and the stable, higher expression of ACE2 compared to the parent Vero cell line make Vero E6 the preferred cell line for plaque-based assays [17,19]. In this paper, the infectivity of the three coronavirus variants, Wild-Type (USA/WA1), Delta (B.1.617.2), and Omicron (B1.1.529) across multiple cell lines was studied. Supernatants from replicate infected wells at 5 days post-infection were processed to quantify viral litres using RT-qPCR, where lower Cq numbers represent high viral litres and cell viability by lactate dehydrogenase activity (LDH).

**Fig. 1.**
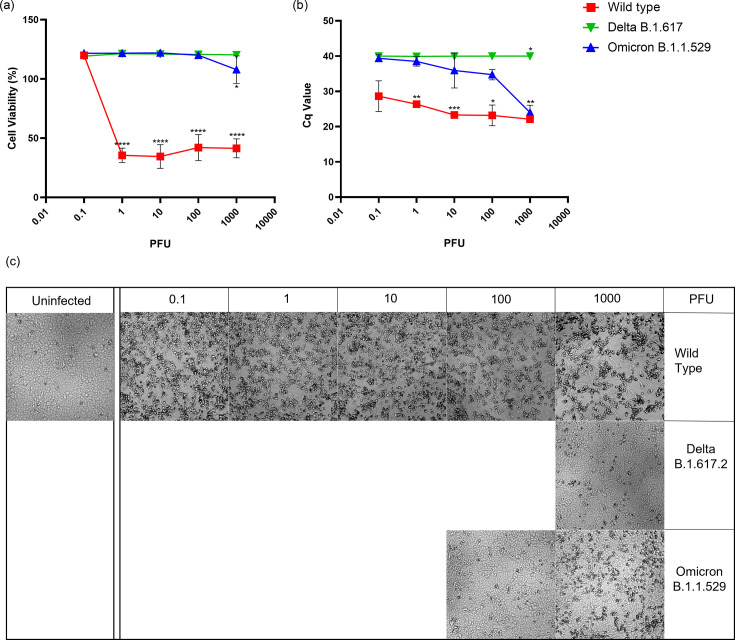
Infection of VeroE6 cells with Wild-Type, Delta and Omicron strains. (**a**) Percent cell viability of infected cells calculated using LDH released in the supernatant from triplicate replicates. All values are normalized to uninfected cell controls. (**b**) Cycle quantification (Cq) values measured by RT-qPCR in supernatant from infected cells 5 days post-infection. (**c**) Monolayer viability was confirmed by visual inspection of cultures by brightfield microscopy. Images from monolayers shown bracket the CPE breakpoints at the PFU indicated indicated * Indicates *p* value≤0.05.

As expected, wild-type SARS-CoV-2 destroyed VeroE6 monolayers at viral doses of 1 PFU to 1000 PFU (Fig. 1c). The WT virus at a dose as low as 1 PFU resulted in a maximum loss of cell viability while producing similar amounts of viral genome across all infection doses tested (Fig. 1a, b). Interestingly, for the lowest viral dose of 0.1, the amount of virus produced and the visual appearance of disrupted monolayers was similar to those at higher PFUs, however, was not detected by LDH release. In contrast, Omicron and Delta variants failed to reduce host cell viability and virus titre increased only for Omicron at the highest PFU tested. It is of note that Omicron litres were equivalent to WT at the highest PFU tested without an appreciable increase in host cell death. Microscope images in Fig. 1c correlate with cell viability in Fig. 1a, where lower cell viability can be observed as CPE or holes in the monolayer.

**Fig. 2.**
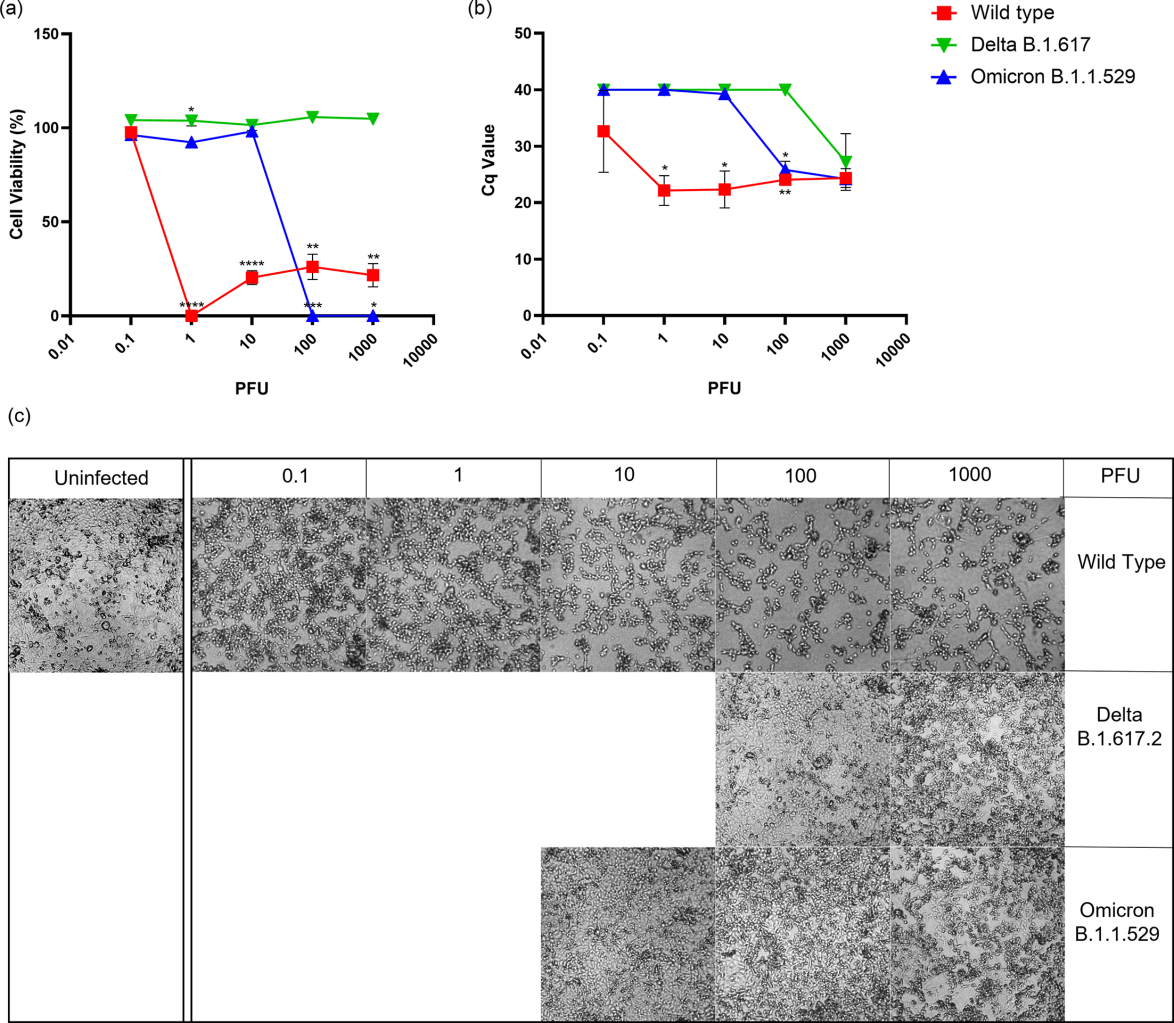
Infection of VeroE6 cells highly expressing endogenous ACE2 with Wild-Type, Delta, and Omicron strains. (**a**) Percent cell viability of infected cells calculated using LDH released in the supernatant from triplicate replicates. All values are normalized to uninfected cell controls. (**b**) Cycle quantification (Cq) values measured by RT-qPCR in supernatant from infected cells 5 days post-infection. (c) Monolayer viability was confirmed by visual inspection of cultures by brightfield microscopy. Images of cultures that bracket CPE breakpoints are shown. (*P<0.05, **P<0.01, ***P<0.001).

Angiotensin Converting Enzyme 2 (ACE2) is a protein found on the surface of most cells and serves as a receptor for SARS-CoV-2 entry [19]. Employing an identical experimental setup to that of Vero E6 cells shown in Fig. 1, VeroE6 high expressing endogenous ACE2 (VeroE6/ACE2) were infected with all three variants (Fig. 2). As in VeroE6 in Fig. 1, for WT SARS-CoV-2, all infection doses reduced cell viability and increased viral litres except the lowest dose that did not lower the cell viability in VeroE6/ACE2 (Fig. 2a, b). Interestingly, the cells infected with the lowest infection dose of WT showed some rounded cells despite the high cell viability (Fig. 2c). Delta variant failed to reduce cell viability and increase viral litres at any infection dose except the highest viral dose 1000 PFU where high viral litres and CPE was observed (Fig. 2). The Omicron variant significantly reduced the viability cells at the two highest viral doses where CPE was also observed (Fig. 2c), 100 and 1000 PFU, which corresponded with viral litres produced as well (Fig. 2b).

**Fig. 3.**
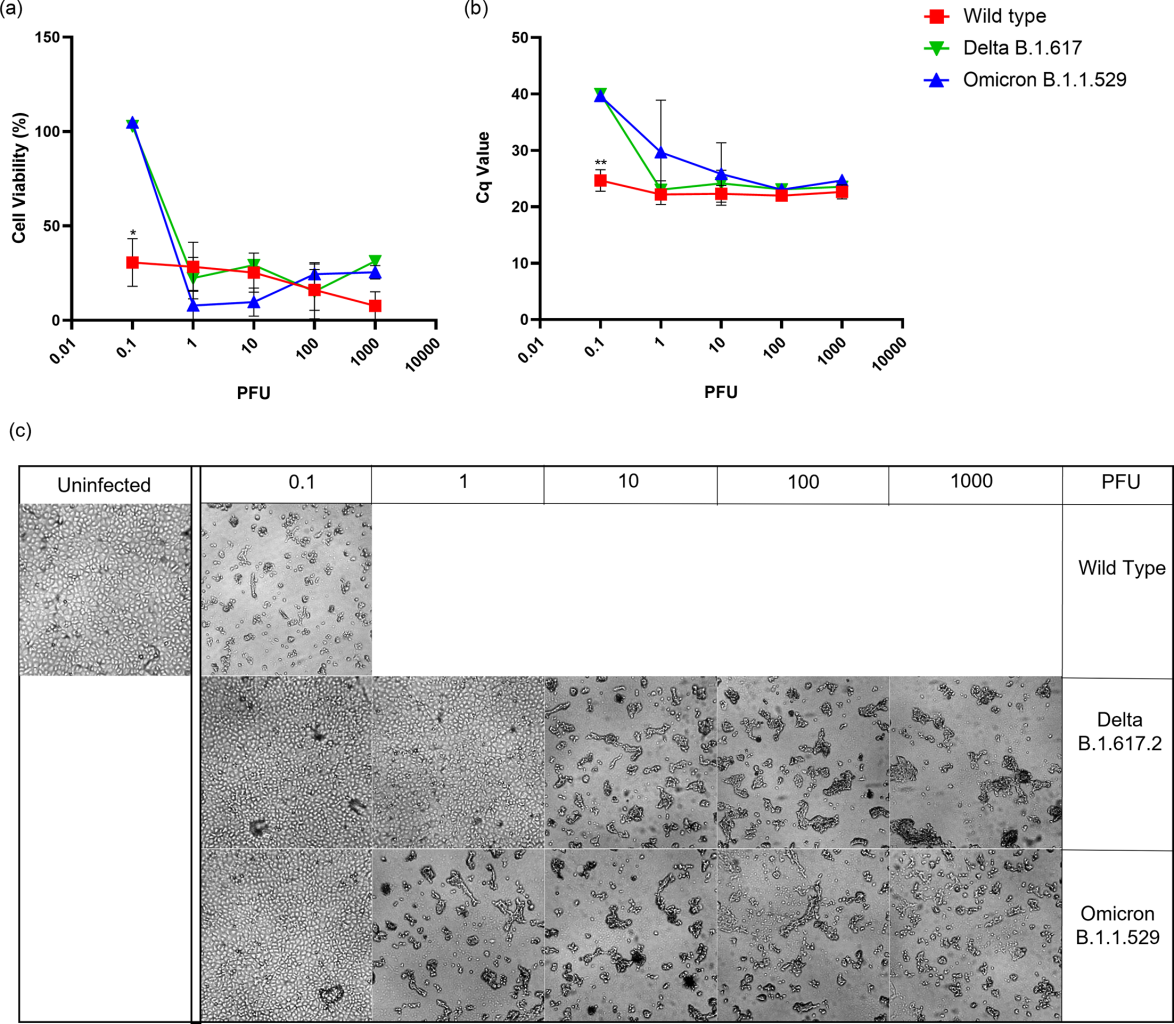
Infection of VeroE6 cells highly expressing human ACE2 and TMPRSS2 with Wild-Type, Delta and Omicron strains. (**a**) Percent cell viability of infected cells calculated using LDH released in the supernatant from triplicate replicates. All values are normalized to uninfected cell controls. (**b**) Cycle quantification (Cq) values measured by RT-qPCR in supernatant from infected cells 5 days post-infection. (**c**) Monolayer viability was confirmed by visual inspection of cultures by brightfield microscopy. Images from monolayers shown bracket the CPE breakpoints at the PFU indicated indicated. (*P<0.05, **P<0.01, ***P<0.001).

The serine protease TMPRSS2 is important for S protein priming during viral entry [13]. When VeroE6 expressing human ACE2 and TMPRSS2 (VeroE6/hACE2/TMPRSS2) were infected with a viral titration of WT strain, all tested infection doses reduced the cell viability, showed high CPE and high viral litres (Fig. 3). On the other hand, both Delta and Omicron reduced cell viability and had high viral litres at all infection doses except the lowest infection dose 0.1 (Fig. 3a, b). CPE caused by Omicron infection followed the same trend where no CPE was observed at the lowest infection dose. In contrast, even with low cell viability and high viral litres at 1 PFU of Delta variant, CPE was not observed (Fig. 3c).

**Fig. 4.**
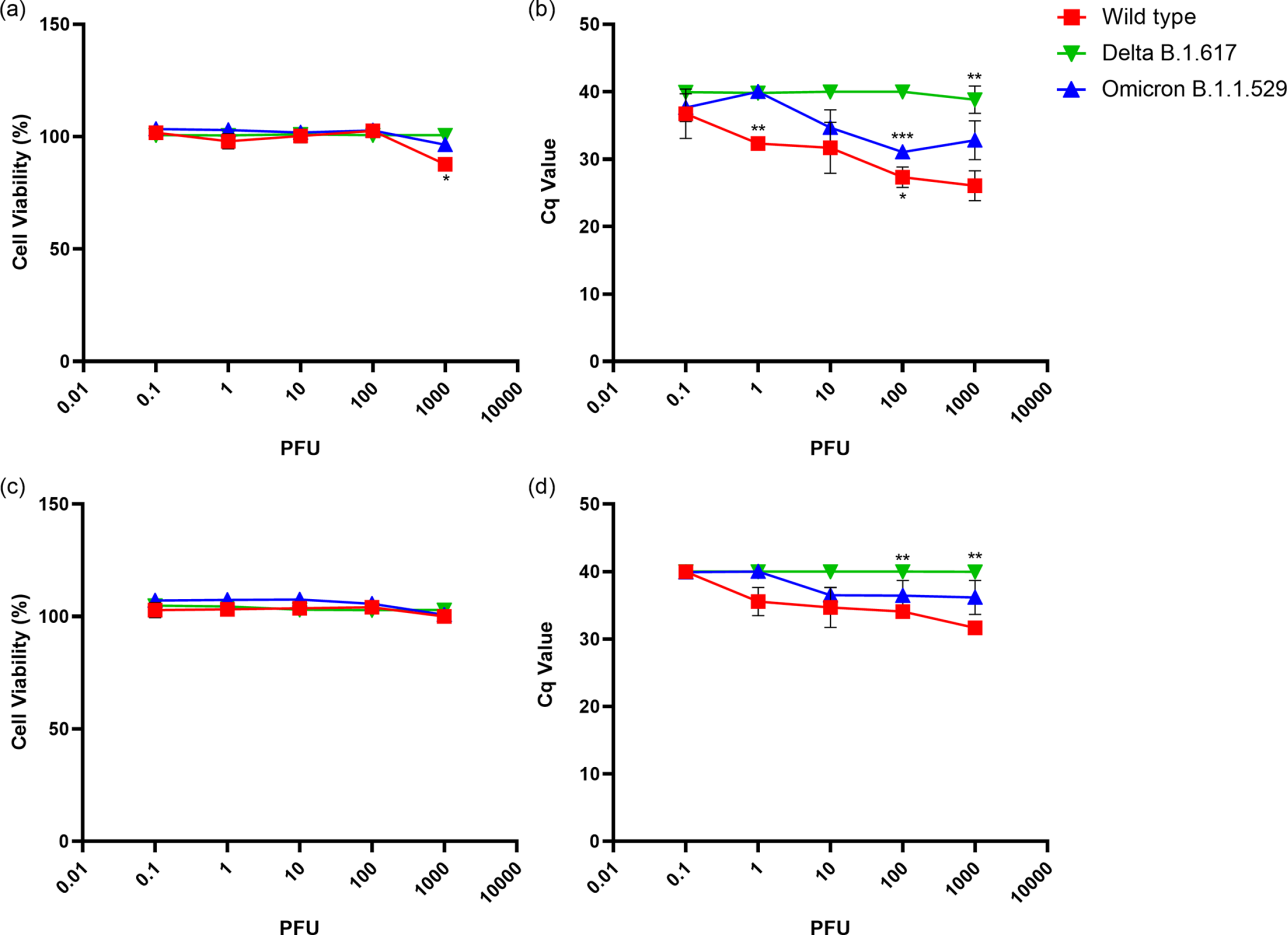
Infection of Calu3 cells highly expressing human ACE2 (Calu3/hACE2) and A549 cells with Wild-Type, Delta and Omicron strains. (**a**) and (c) Percent cell viability of infected Calu3/hACE2 and A549 cells calculated using LDH released in the supernatant from triplicate replicates, respectively. All values are normalized to uninfected cell controls. (**b**) and (d) Cycle quantification (Cq) values measured by RT-qPCR in supernatant from infected Calu3/hACE2 and A549 cells 5 days post-infection, respectively. (*P<0.05, **P<0.01, ***P<0.001).

Human lung cell lines, Calu3 high expressing ACE2 (Clu3/Hace2) and A549 (Fig. 4) were infected with the three SARS-CoV-2 variants. No significant reduction in cell viability was measured (Fig. 4a, c) at any infection dose for all three variants. Viral litres measured after RT-qPCR were dose dependent for WT and Omicron infected lung cells. Specifically for Omicron, an increase in viral litres was observed at infection dose 10 PFU. For cells infected with Delta, low viral litres were observed at all infection doses (Fig. 4b, d) in both cell lines.

## Discussion

There is extensive ongoing research on the different variants of SARS-CoV-2 that have emerged in the last 3 years. Each variant with its unique infection kinetics and pathogenicity is investigated in numerous different models. Having a defined cell culture model for all the variants is critical for *in vitro* studies, such as neutralization assays or antiviral testing. Understanding the infection kinetics of different variants in the same environment will not only give us a better understanding of the viral pathogenesis but also help us choose the right infection model for *in vitro* experiments.

The viral spike protein binds to the ACE2 receptor on cells and uses TMPRSS2 present on the host cell membrane or cathepsins present in the endosome to cleave the S protein [13]. The contents of the virus are transferred into the cytoplasm of the cell where the host cell machinery can be used for viral replication. The viral particles produced are released through lysosomal exocytosis into the extracellular matrix [20]. SARS-CoV-2 infected cells have Spike protein on their surface that can bind to the ACE2 receptor on adjacent uninfected cells that can cause cell-cell fusion called syncytia [21]. Syncytia formation not only helps the virus evade the immune system and spread to more cells but also can make the cells more prone to cell death [22]. Other ways a SARS-CoV-2 infection can induce cell death or CPE are apoptosis, autophagy, necroptosis, and inflammation activation in the host cells [23].

We have highlighted two important parameters to consider when selecting a cell culture model; CPE and replication. CPE is measured by measuring the cell viability of the infected cells using lactate dehydrogenase (LDH) released in the media. LDH is released after a cell has succumbed to an infection. CPE can be directly correlated to the amount of LDH present in the cell culture media which can be corroborated visually under a microscope. Cells that undergo CPE, start rounding up and die leaving holes in the monolayer. SARS-CoV-2 virions released in the supernatant of infected cells were quantified using RT-qPCR. The increase in RNA copies (inversely related to Cq value) in the supernatant, correlates with viral replication.

Both Omicron and Delta replication was attenuated compared to WT in VeroE6 cells at 120 h post-infection (Fig. 1b) which was also observed at 24 and 48 h post-infection [24]. At 72 h post-infection, Omicron and Delta had comparable viral replication in VeroE6 cells at 0.1 infection dose [25], which was also observed at 120 h post-infection (Fig. 1b). However, when the infection dose is increased to 10 PFU – 1000 PFU, Omicron shows higher replication than Delta in VeroE6 cells (Fig. 1b). Both Omicron and Delta failed to cause a reduction in cell viability of VeroE6 cells at all the infection doses tested, except 1000 PFU for Omicron that produced a noticeable CPE when observed under the microscope (Fig. 1c).

When ACE2 is overexpressed in VeroE6 cells, Omicron variant replication increased significantly, which was not observed for Delta (Fig. 2). Similarly, with the higher expression of ACE2 in Calu3 cells, viral release in the supernatant of Calu3/hACE2 cells infected with Omicron strain was higher than that of Delta at day five post-infection (Fig. 4), compared to Calu3 cells where Omicron has shown to have less viral particle release than Delta at 2 days post-infection [23,25]. In both Calu3 and VeroE6, overexpression of ACE2 increased infectivity of Omicron suggesting an increased importance of ACE2 for the pathogenesis of Omicron compared to Delta. This could be due to Omicron spike protein having a higher binding affinity to ACE2 compared to Delta spike protein as shown in previous studies [26,29]. Even though Omicron spike protein binding affinity is higher than WT, the inherent susceptibility of VeroE6 cells to WT (Fig. 1) is responsible for the high infectivity of WT in VeroE6/ACE2 cells.

Replication of Omicron in VeroE6/ACE2 cells was higher than Delta, but when TMPRSS2 was overexpressed, replication of both the variants was comparable (Figs 2 and 3). This suggests that the presence of TMPRSS2 has a larger impact on Delta replication than it does on Omicron replication. Consistent with previous studies [24,25, 30], Omicron utilizes TMPRSS2 less efficiently for S cleavage than Delta or WT and uses the endocytic pathway instead for cell surface entry [31].

At 120 h post-infection the WT strain shows dose dependent CPE in VeroE6 but no CPE in Calu3/hACE2 or A549s (Fig. 4) [32]. Omicron and WT viral litres in the supernatants of Calu3/hACE2 cells were significantly higher than Delta. Cell lysates of Calu3/hACE2 have shown higher viral litres for Delta compared to Omicron [33] suggesting an altered viral release of Delta from Calu3/hACE2. Similarly, in A549, no CPE was observed at day 5 for all three variants with minimal viral release observed for WT and Omicron. ACE2 and TMPRSS2 expression in A549 cells is less than in other cell lines [34,35] thus contributing to the low infectivity of A549 cells. Although the WT passages retained virulence in animals, it is possible that minor polybasic mutations could contribute the lack of CPE in the Calu-3 cell with virus replication (Fig. 4).

In conclusion, for CPE or plaque-based assays, such as neutralization assays or antiviral testing assays, choosing VeroE6/hACE22/TMPRSS2 would be beneficial over VeroE6 given the former cell lines’ higher sensitivity to SARS-CoV-2 infection. This is a unique study with a parallel comparison of multiple variants across multiple cell lines that highlights the importance of understanding cell line sensitivities to different variants that not only contributes towards future experimental design but also towards understanding the pathogenesis of the variants.

## Abbreviations

ACE: angiotensin-converting enzyme
CPE: cytopathic effects
CQ: cycle quantification
LDH: lactate dehydrogenase
RT-PCR: reverse transcriptase-polymerase chain reaction
TMPRSS: trans-membrane serine protease
WT: wild-type
